# Precometary organic matter: A hidden reservoir of water inside the snow line

**DOI:** 10.1038/s41598-020-64815-6

**Published:** 2020-05-08

**Authors:** Hideyuki Nakano, Naoki Hirakawa, Yasuhiro Matsubara, Shigeru Yamashita, Takuo Okuchi, Kenta Asahina, Ryo Tanaka, Noriyuki Suzuki, Hiroshi Naraoka, Yoshinori Takano, Shogo Tachibana, Tetsuya Hama, Yasuhiro Oba, Yuki Kimura, Naoki Watanabe, Akira Kouchi

**Affiliations:** 1 Department of Science, Kyoto University of Education, 1 Fukakusafujinomori-cho, Fushimi-ku, Kyoto, 612-8522 Japan; 2 Institute for Planetary Materials, Okayama University, 827 Yamada, Misasa, Tottori, 682-0193 Japan; 3 Research Institute for Geo-resources and Environment, Geological Survey of Japan, National Institute of Advanced Industrial Science and Technology, Tsukuba, Ibaraki, 305-8567 Japan; 4 Department of Earth and Planetary Sciences, Hokkaido University, Sapporo, Hokkaido, 060-0810 Japan; 5 Department of Earth and Planetary Sciences/Research Center for Planetary Trace Organic Compounds, Kyushu University, 744 Motooka, Nishi-ku, Fukuoka, 819-0395 Japan; 6 Biogeochemistry Program, Japan Agency for Marine-Earth Science and Technology, 2-15 Natsushima, Yokosuka, Kanagawa 237-0061 Japan; 7 UTokyo Organization for Planetary and Space Science, University of Tokyo, 7-3-1 Hongo, Tokyo, 113-0033 Japan; 8 Japan Aerospace Exploration Agency, Sagamihara, Kanagawa 252-5210 Japan; 9 Institute of Low Temperature Science, Hokkaido University, Sapporo, Hokkaido 060-0819 Japan; 10 Present Address: Department of Sport Education, Toin University of Yokohama, 1614 Kurogane-cho, Aoba-ku, Yokohama, 225-8503 Japan; 11 Present Address: Graduate School of Engineering Science, Yokohama National University, 79-5 Tokiwadai, Hodogaya-ku, Yokohama, 240-8501 Japan; 12 Present Address: Ube Industries, Ltd., 1978-96 Kogushi, Ube, Yamaguchi, 755-8633 Japan

**Keywords:** Asteroids, comets and Kuiper belt, Early solar system

## Abstract

The origin and evolution of solar system bodies, including water on the Earth, have been discussed based on the assumption that the relevant ingredients were simply silicates and ices. However, large amounts of organic matter have been found in cometary and interplanetary dust, which are recognized as remnants of interstellar/precometary grains. Precometary organic matter may therefore be a potential source of water; however, to date, there have been no experimental investigations into this possibility. Here, we experimentally demonstrate that abundant water and oil are formed via the heating of a precometary-organic-matter analog under conditions appropriate for the parent bodies of meteorites inside the snow line. This implies that H_2_O ice is not required as the sole source of water on planetary bodies inside the snow line. Further, we can explain the change in the oxidation state of the Earth from an initially reduced state to a final oxidized state. Our study also suggests that petroleum was present in the asteroids and is present in icy satellites and dwarf planets.

## Introduction

Water plays an essential role in various hydrological, geological, and chemical processes in terrestrial planets and is key to discussions of the origins of life and biological activity. Despite its obvious importance, the origin of water on Earth is not fully understood^1–4^. Recently, analyses of some isotopes from the comet 67 P/Churyumov–Gerasimenko have shown that the contribution of cometary ice to the Earth’s oceans is less than 1%^5^, indicating the necessity for other candidates for the source material of terrestrial water. To deliver water to terrestrial-planet embryos, various sources have been proposed, *e.g*., the adsorption of water vapor on anhydrous silicates^6^, the incorporation of inward-drifting hydrous silicate grains into planetesimals at approximately 1 AU^7^, and ice-pebble accretion due to the migration of the snow line^8^. However, most models fail to explain the fact that the initially accreted Earth was a highly reduced environment^9,10^. In the late-stage water delivery models known as the classical^11^ and Grand Tack^12,13^ models, one problem is that too much water is delivered to the terrestrial planets if it is delivered as hydrous silicates in carbonaceous chondrites from beyond the snow line.

In studies of the origin of water on Earth, much less attention has been paid to “precometary organic matter,” *i.e*., abiotic hydrocarbons with oxygen-containing functional groups and their derivatives in space, as opposed to ices and silicates. However, astrophysical observations indicate that organic molecules are ubiquitous in space. For example, the space probes used in the missions to the comets 1 P/Halley and 67 P/Churyumov–Gerasimenko have shown that the main components of cometary refractory grains are organic matter and silicates^14,15^. Further, analyses of interplanetary dusts^16,17^, cometary dusts collected from the comet 81 P/Wild 2^17,18^, and micrometeorites^19^ have shown that organic refractory grains are also abundant and ubiquitously distributed in these extraterrestrial materials. Many laboratory experiments suggest that these organic substances are formed in interstellar molecular clouds^20^ and then processed in the outer parts of the solar system^21^ via chemical processes such as ultraviolet photolysis, heating, and even the quantum tunneling of simple atoms and molecules^22^. These studies suggest that the abundant organic matter in space is of particular importance in assessing the origin and evolution of solar system bodies.

To investigate the distribution of precometary organic matter in the solar nebula, researchers have conducted heating experiments on precometary-organic analogs^23–25^. Nakano *et al*.^25^ demonstrated that fresh precometary organics evaporate at temperatures between 200 K and 300 K and that carbonaceous materials persist at temperatures higher than 300 K. The distribution of organics in the solar nebula can be determined from this result if we assume a suitable temperature distribution for the solar nebula. These organic-covered silicate grains accreted together with chondrules and other minerals in the solar nebula to form the meteoritic parent bodies. Nakano *et al*.^26^ performed experiments on the reactions of precometary organics with water followed by heating in a vacuum using an precometary-organic analog to mimic aqueous alteration and thermal metamorphism in the parent bodies of carbonaceous chondrites. They found that O and N-rich viscous organic matter obtained by aqueous alteration changed to solid carbonaceous materials after heating in a vacuum. However, there have been no experimental investigations with respect to the heating of precometary organics in parent bodies inside the snow line.

In the present investigation, we experimentally studied the potential role of precometary organic matter as a source of water inside the snow line (<2.5 AU) by simulating the heating of organic matter up to several hundreds of degrees Kelvin in the parent bodies of ordinary chondrites^27–29^. Under such high-temperature conditions, the complex organic molecules present in the parent bodies can decompose into small molecules, including water, even inside the snow line, and the produced water may serve as a source material for terrestrial water.

## Chemical Compositions of Interstellar-Organic Analogs

Large amounts of precometary organic matter cannot be obtained; therefore, we used chemical reagents to create an analog for precometary organic matter that formed in a molecular cloud^24,25^. The chemical compositions of organic matter produced by the photolysis of an ice mixture depends on the composition of the ice mixture^30–32^. Briggs *et al*. used a CO-rich ice mixture (H_2_O:CO:NH_3_ = 5:5:1) for photolysis experiments^30^, while Bernstein *et al*. used a CH_3_OH-rich ice mixture (H_2_O:CH_3_OH:CO:NH_3_ = 10:5:1:1)^32^. Infrared observations^33,34^ indicate the presence of CO-rich ices in low-mass protostars similar to our Sun and in molecular clouds. Further, the compositions of cometary ices, which are considered to be remnants of interstellar ices, are CO-rich and CH_3_OH-poor^35^. Conversely, CH_3_OH-rich ices are observed primarily in high-mass protostars. Because CH_3_OH is formed by the hydrogenation of CO in molecular clouds^36^, one can reasonably expect that the hydrogenation of CO proceeded only to a certain extent (forming only a few percent CH_3_OH) in the molecular cloud where our solar system was born. Therefore, the ice mixture used by Briggs *et al*.^30^ is more relevant for discussions of the organic matter in our solar system than that used by Bernstein *et al*.^32^. Furthermore, although the ice-composition dependence of the organic residues’ composition has been previously investigated^37,38^, a recipe based on these works is precluded by the deficiency of analytical data.

**Figure 1:**
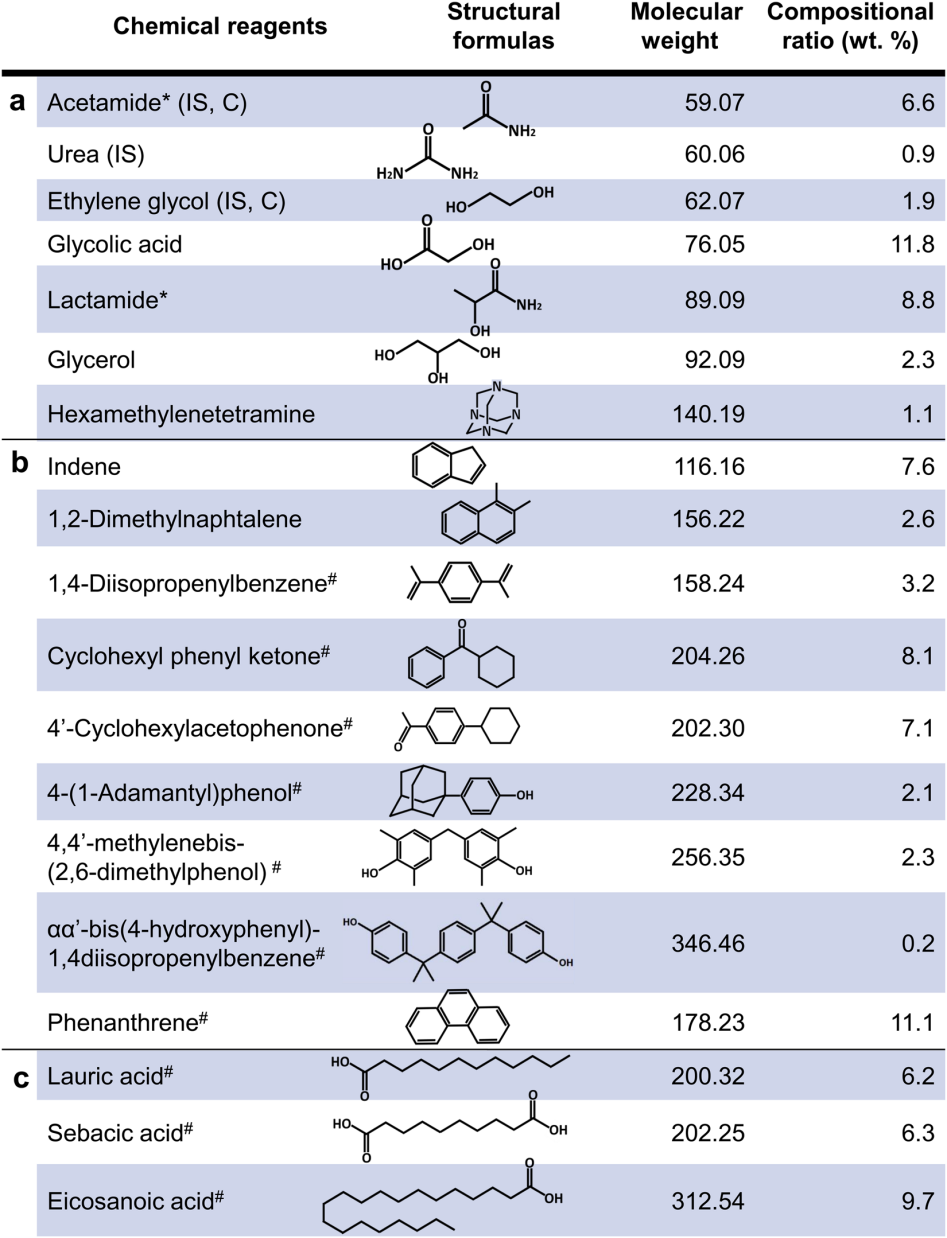
Composition of the precometary-organic-matter analog (MC). To formulate the recipes in groups a, b, and c, we referred to the analytical data in refs. ^30,39,40^, respectively. *When we could not obtain the chemical reagents from the analyses, we used reagents with similar chemical characteristics. ^#^Unidentified chemical compounds were estimated from their elemental compositions. IS and C indicate chemical compounds observed in interstellar space^41–44^ and comets^45,46^, respectively.

To formulate the recipe for our analog, we referred to analytical data^30,39,40^ produced by the photolysis of mixed ices (H_2_O:CO:NH_3_ = 5:5:1). Briggs *et al*. analyzed soluble organic matter using gas chromatography-mass spectrometry (GC-MS) and showed that the lower molecular weight compounds were C_2_–C_3_ hydroxy acids and hydroxy amides, glycerol, urea, hexamethylenetetramine, and formamidine^30^. Greenberg and Mendoza-Gomez analyzed the less soluble part of the same sample using high-resolution fast atom bombardment mass spectrometry and proposed the following elemental compositions: C_9_H_8_, C_12_H_12_, C_12_H_14_, C_13_H_16_, C_13_H_16_O, C_14_H_18_O, and C_14_H_18_O_2_ ^39^. Further analyses of the same sample using electron impact mass spectrometry indicated that the sample consists of a complex mixture of hydrocarbon chains (-CH_2_-)_n_ from C_5_ to C_30_ ^40^. Figure 1 shows the recipe used in the present study, called molecular cloud (MC) organics. Some of the compounds in this recipe have been found in interstellar space^41–44^ and comets^45,46^. The chemical compounds in Fig. 1a,b, and 1c are based on the analytical data of Briggs *et al*.^30^, Greenberg and Mendoza-Gomez^39^, and Mendoza-Gomez and Greenberg^40^, respectively. We used reagents with similar chemical characteristics when we could not obtain the same chemical compounds as in the analyses, and we estimated unidentified chemical compounds from their elemental compositions. In addition, to discuss the formation mechanism of water, we used two specific compositions: alcohols and carbonic acid (Fig. SI 1a) and ketone and amides (Fig. SI 1b).

## Results

### *In-situ* observations using a diamond anvil cell

**Figure 2:**
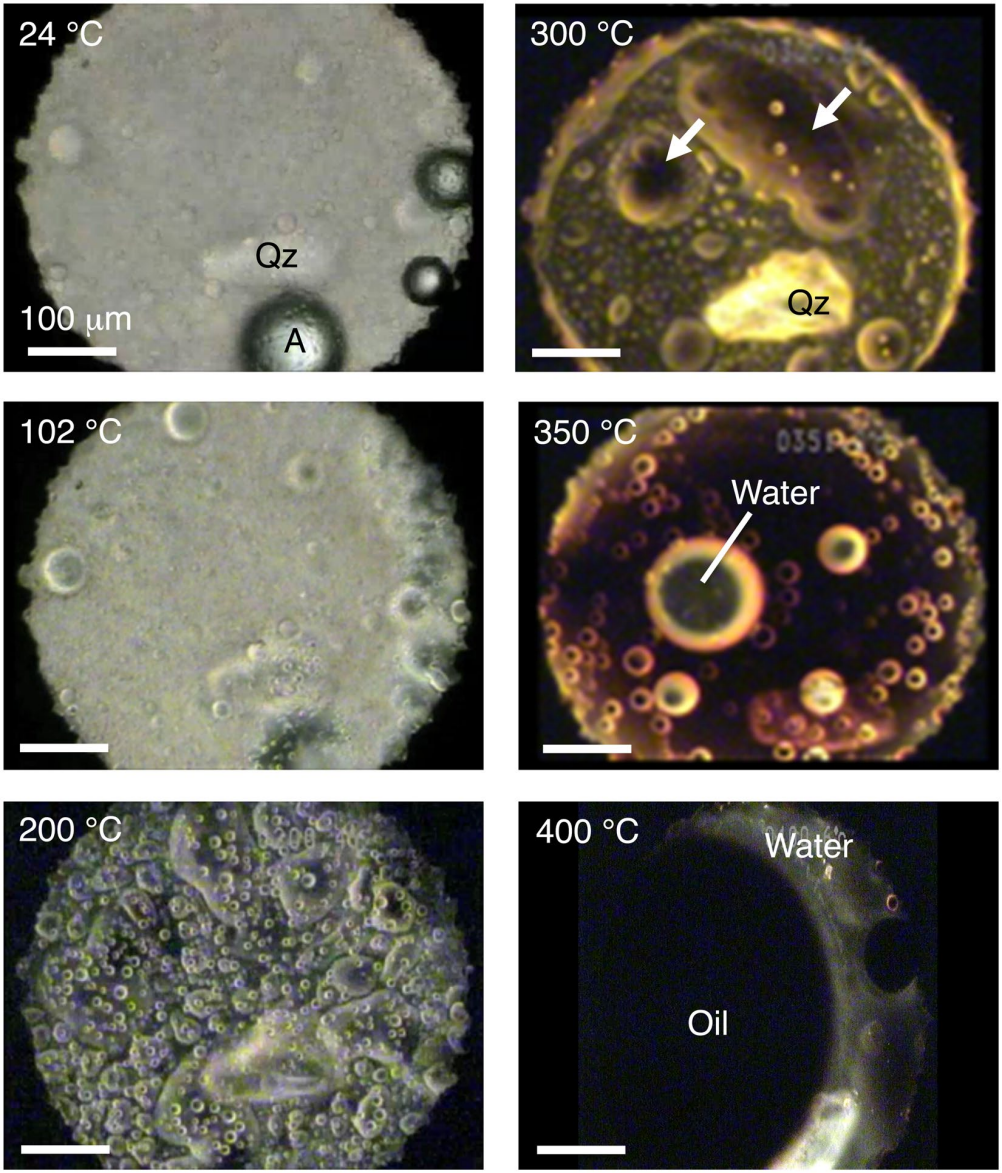
*In-situ* observations of the heating of an precometary-organic-matter analog (MC) using a diamond anvil cell. Photomicrographs were taken during the heating experiment. A indicates air bubbles, and Qz indicates quartz crystals. White arrows indicate highly viscous organic matter. The scale bars represent 100 μm.

**Figure 3:**
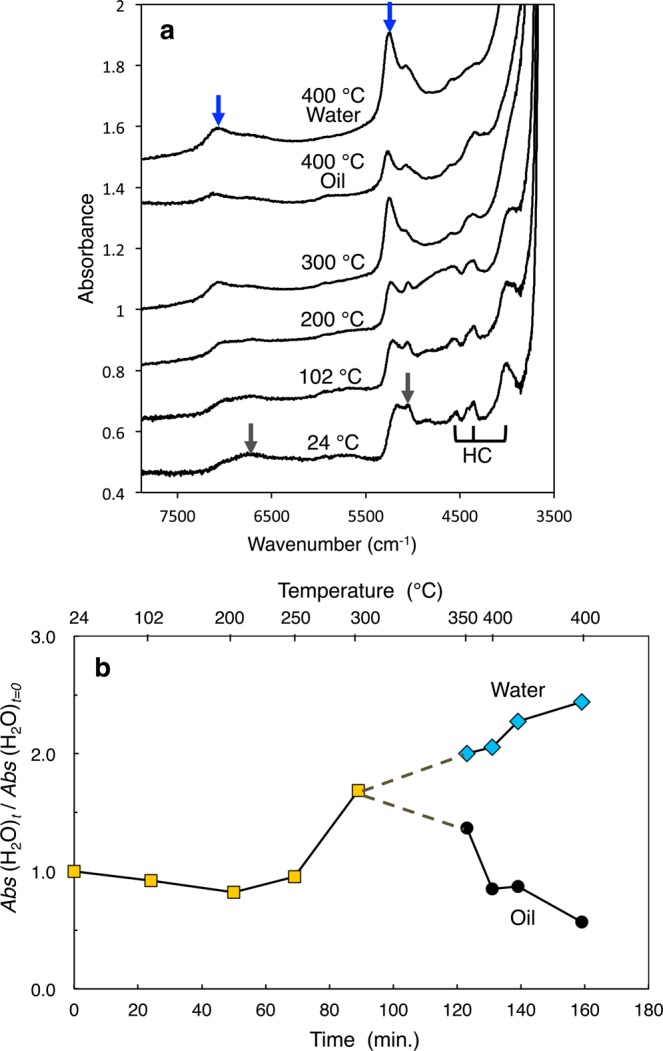
(**a**) Near-infrared absorption spectra of the precometary-organic-matter analog (MC) at temperatures between 24 °C and 400 °C. Blue arrows indicate absorption bands owing to H_2_O, gray arrows indicate absorption bands owing to OH, amine, and ammonia (5000 cm^−1^) and amine and ammonia (6800 cm^−1^), and HC (3991, 4364, and 4530 cm^−1^) indicates hydrocarbons. (**b**) The ratio of the 5200-cm^−1^ water band peak height at time *t* to that at *t* = 0. The upper abscissa shows the corresponding temperatures.

To study the heating of organic matter, we first made *in-situ* observations using an externally heated diamond anvil cell^47^ with the temperature profile shown in Fig. SI 2. Figure 2 and Videos SI 1–SI 7 show photomicrographs and videos, respectively, that we obtained during the heating experiments. At temperatures between 24 °C and 150 °C, the sample was uniform, and the color changed from pale yellow to yellow. At approximately 150 °C, phase separation occurred and two organic phases coexisted until 350 °C. One was transparent and had low viscosity, while the other was dark brown and had high viscosity. The latter is hereafter referred to as “highly viscous organic matter.” The viscosity of the highly viscous organic matter increased with the temperature. At approximately 350 °C, the two organic phases became one phase again, the color changed to reddish-black, and the viscosity decreased. From these changes in the color and viscosity, we concluded that polymerization proceeded until 350 °C and that thermal cracking occurred at temperatures higher than 350 °C. This process is nearly the same as the process that forms geopolymer kerogen and subsequently petroleum^48^. Water droplets formed simultaneously at approximately 350 °C, and the droplets coalesced as temperature rises. At 400 °C, water and black oil coexisted, and they continued to coexist after the sample had been cooled to room temperature. Figure 3a shows the change in the near-infrared spectra with temperature. At temperatures between 24 °C and 200 °C, the sample has rich functional groups, including -CH_2_, -CH_3_, -OH, and -NH_2_. At temperatures higher than 300 °C, two absorption bands originating from water are clearly observed; the bands at 5200 cm^−1^ and 7100 cm^−1^ are due to a combination of OH stretching and bending vibrations and to the OH stretching overtone of water, respectively^49^. Meanwhile, the presence of -CH_2_, -CH_3_, and -NH_2_ became unclear due to the strong H_2_O bands. At 400 °C, the difference between the oil and the water became clearer. Figure 3b shows the change in the intensity of the 5200-cm^−1^ water band with time (*i.e*., temperature), clearly showing that the intensity of water increased at approximately 300 °C. Therefore, we concluded that water formation started at approximately 300 °C. Note that this conclusion is valid only for the laboratory time scale of heating. On astrophysical time scales, the water formation temperature should be lower than 300 °C.

## Reactor Experiments

**Figure 4:**
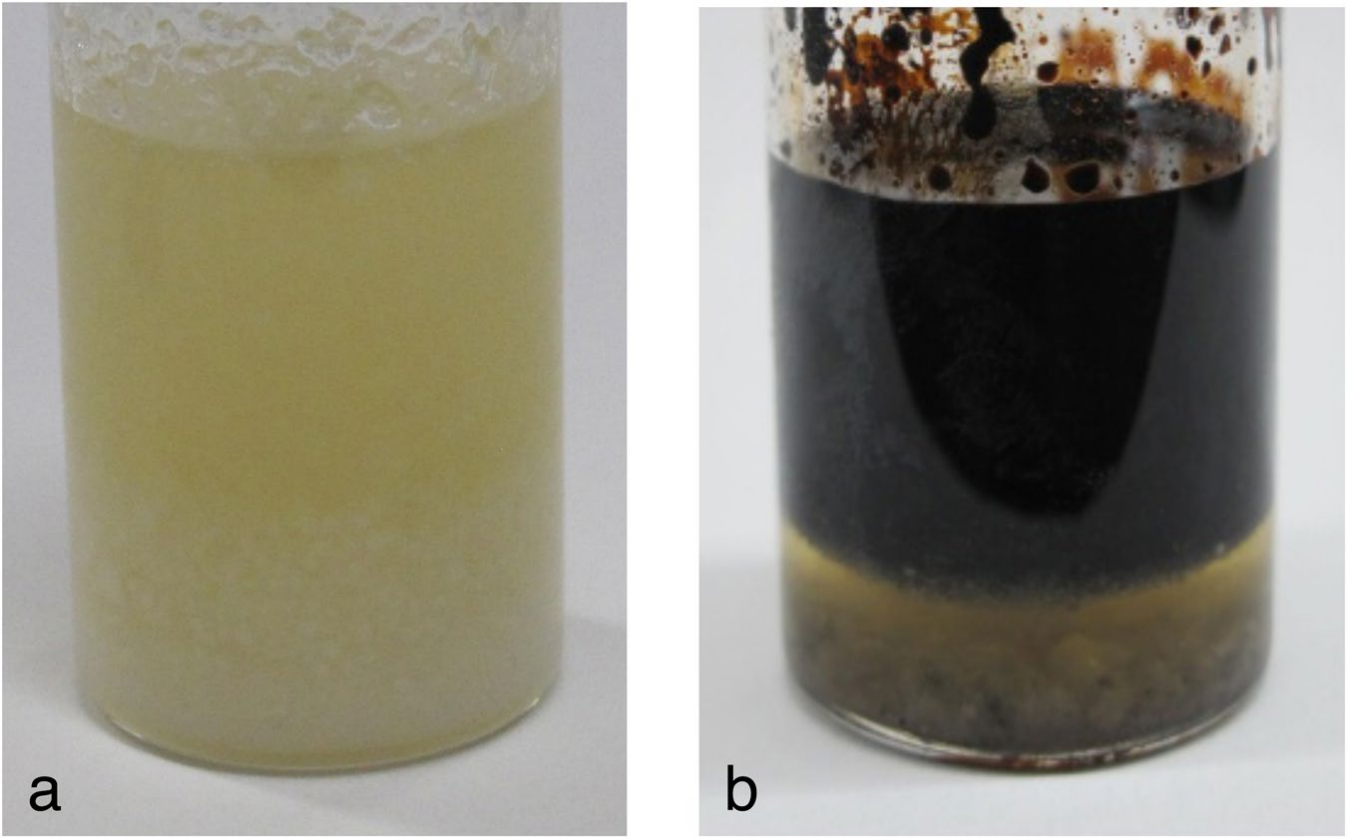
Photographs of **(a)** the starting material, *i.e*., the precometary-organic-matter analog (MC) shown in Fig. 1, and **(b)** the black oil and aqueous products recovered from the MC heating experiment at 400 °C. The diameter of the sample bottle is 30 mm.

To characterize the water and black oil produced in this experiment in more detail, we also performed heating experiments using an autoclave, as shown in Fig. SI 3. Figure 4a,b show photographs of the starting material and the recovered products after 5 h of heating at 400 °C and 14 MPa, respectively. The color of the aqueous product is a cloudy pale brown, and the oil is black and viscous similar to crude oil. The densities of the oil and the aqueous product measured at 18.4 °C are 997.3 ± 48.2 kg m^−3^ and 1060.7 ± 42.7 kg m^−3^, respectively. The pH of the aqueous product was 9.3.

**Figure 5:**
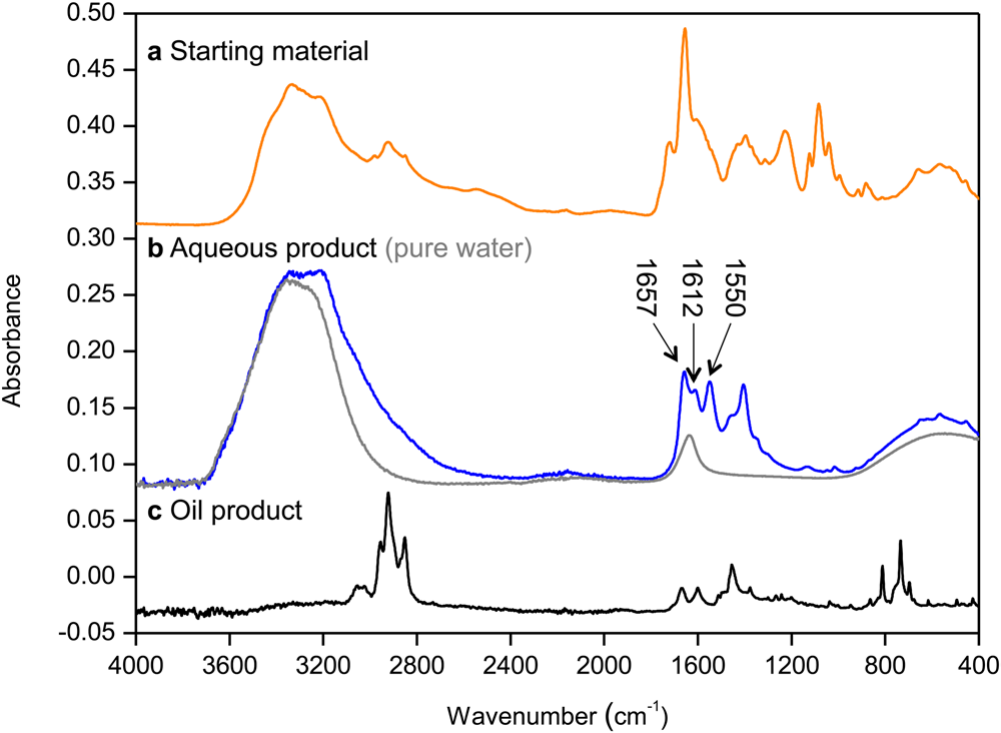
Mid-infrared absorption spectra of **(a)** the starting material (MC); **(b)** the recovered aqueous product, together with pure water; and **(c)** the recovered oil product.

**Figure 6:**
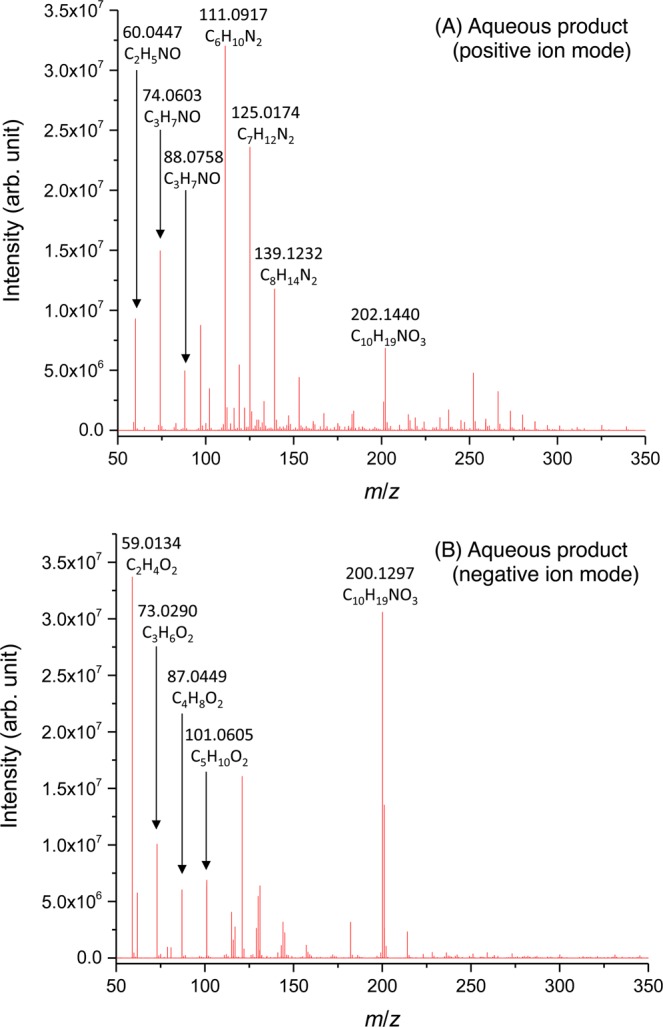
High-resolution mass spectra of the recovered aqueous products from the heating of MC measured in the **(A)** positive- and **(B)** negative-ion modes over an *m*/*z* range of 50–350. We assigned possible molecular formulas to several peaks based on their exact masses.

Figure 5 shows the mid-infrared spectra of the starting material, the aqueous and oil products, and a reference spectrum of pure liquid water. The spectrum of the aqueous products clearly shows the OH stretching–vibration band at approximately 3800−2800 cm^−1^ and the libration band below 800 cm^−1^. These features are in good agreement with those of pure water, indicating that liquid water is the main component of the aqueous product. The OH_2_ bending–vibration band of water [δ(OH_2_)] at approximately 1700–1600 cm^−1^ appears to be buried within strong bands at 1657 cm^−1^, 1612 cm^−1^, and 1550 cm^−1^ due to other products, which can plausibly be attributed to the amide I and II bands and to the antisymmetric COO^−^ stretching–vibration bands of carboxylate anions, respectively^50^. The presence of acetamide (CH_3_CONH_2_ or C_2_H_5_NO), which we used as a reactant, and its alkylated homologues (*e.g*., CH_3_CH_2_CONH_2_ or C_3_H_7_NO), as well as low-molecular weight carboxylic acids such as acetic acid (CH_3_COOH or C_2_H_4_O_2_), propionic acid (CH_3_CH_2_COOH or C_3_H_6_O_2_) and other compounds, are inferred from the high-resolution mass spectrum of the recovered aqueous products (Fig. 6).

**Figure 7:**
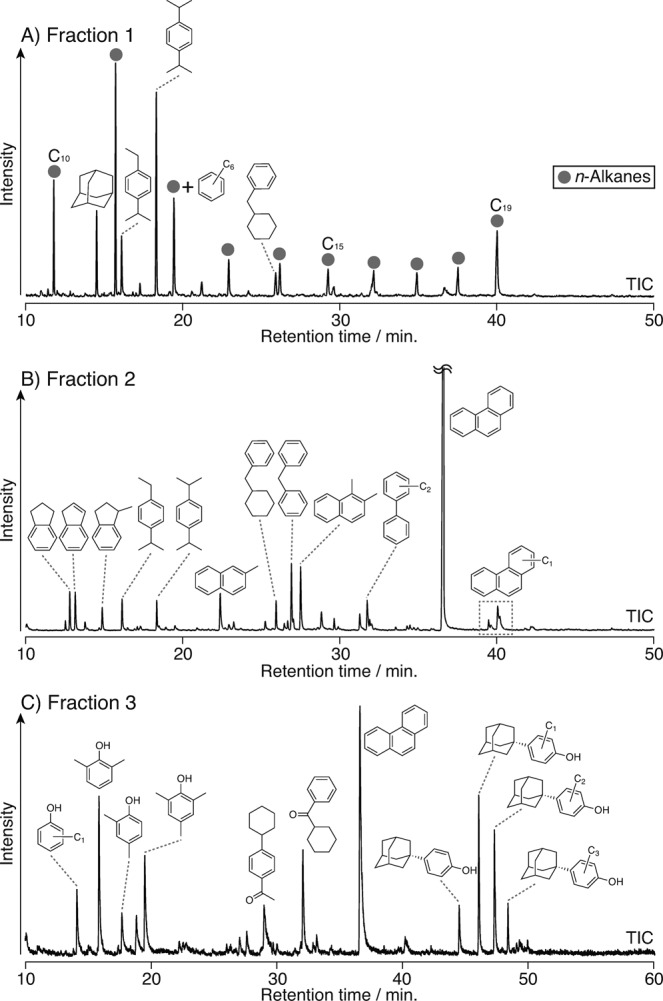
Total-ion-current chromatograms of the recovered oil from the heating of MC: **(A)** fraction 1; **(B)** fraction 2; and **(C)** fraction 3.

Figures SI 4 shows the matrix-assisted laser desorption/ionization (MALDI)-time of flight (TOF) spectrum for the recovered oil during the heating experiment of MC at 400 °C. Many peaks appear in the spectrum, the mass-distribution pattern of which closely resembles that of crude oil^51^. To identify specific molecules in the oil, we subjected the sample to a further series of column chromatography on silica gel. The total-ion-current chromatograms of the recovered oil are shown in Fig. 7a–c. We detected a series of *n-*alkanes (C_10_–C_19_) and alkylated benzenes (C_11_–C_13_) as the main compounds in fraction 1 (Fig. 7a). The *n*-alkanes were generated by decarboxylation and/or the thermal cracking of fatty acids. This does not contradict the absence of COOH bands in the infrared spectrum of the recovered oil (Fig. 5c) because unsaturated hydrocarbons occurred only in trace concentrations in the oil. This result also shows similar characteristics to the components of typical crude oils. Various aromatic compounds were detected in fraction 2 (Fig. 7b). These were likely generated by thermal alterations of starting materials such as methylation, isomerization, and aromatization. The analytical results for fraction 2 are consistent with the mid-infrared absorption spectrum (Fig. 5c). In addition, several aromatic compounds containing hydroxyl or carbonyl groups were found in fraction 3 (Fig. 7c).

**Figure 8:**
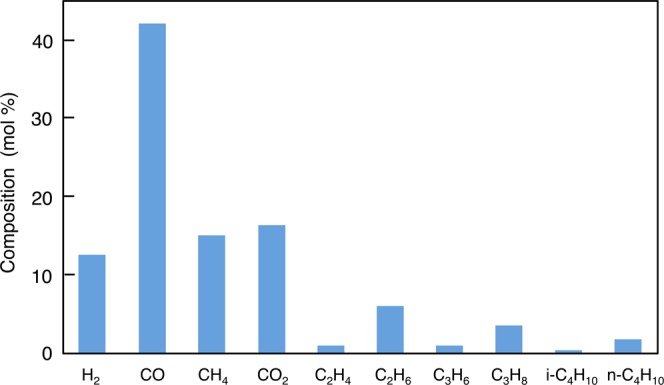
Composition of the recovered gases from the heating of MC.

We detected CO, CO_2_, CH_4_, H_2_, C_2_H_6_, and C_3_H_8_ molecules as major components in the recovered gas (Fig. 8), which shows the significant progress of the decarboxylation and thermal cracking of the C–C bond. Higher concentrations of H_2_ and trace amounts of the unsaturated hydrocarbons C_2_H_4_ and C_3_H_6_ suggest a sufficient generation of H_2_ for the hydrogenation of double bonds. However, we did not detect any nitrogen compounds, because the N-content of MC itself is small and generation of N_2_ from the nitrogen compounds requires a higher heating temperature. Most of the ammonia (NH_3_) generated was dissolved in the aqueous solution as NH_4_^+^.

Pyrolysis experiments for single molecules^52^ suggest that water can be formed by the dehydration–condensation reactions of alcohols and carboxylic acids. To check this hypothesis, we performed further heating experiments using simple mixtures (Fig. SI 1): an -OH and -COOH-bearing mixture called MC-1 and a mixture of amides and ketones without -OH and -COOH called MC-2. Figures SI 5a,b show photographs of the recovered products heated to 400 °C, showing that the recovered products are black oil and a relatively transparent liquid. The weight ratios of the gas to the recovered samples in MC-1 and MC-2 were 72:28 and 25:75, respectively, and those of the black oil to the transparent liquid in MC-1 and MC-2 were 6:22 and 52:23, respectively. Figure SI 6 shows infrared spectra of pure water and the recovered transparent products. The spectrum of the sample obtained from MC-1 is nearly the same as that of pure water, confirming that the main component of the transparent product is water. Conversely, the spectrum of the sample obtained from MC-5 differs greatly from that of pure water but resembles that from MC (Fig. 6b). The peak height ratio of the spectrum at 3340 cm^−1^ to that at 1391 cm^−1^ of the samples from MC-1, MC, and MC-2 are 5.6, 2.3, and 0.45, respectively. These two peaks were selected because 3340 cm^−1^ reflects the amount of -OH and because 1391 cm^−1^ was the strongest peak in the products from MC-2. The OH-contents, defined as the ratio of the OH weight to the total sample weight, of the starting materials of MC-1, MC, and MC-2 were 47.8%, 8.3%, and 0%, respectively. These results clearly indicate that results of pyrolysis experiments for single molecules^52^ provide useful guidelines for water formation from mixed samples and that the amount of water formed depends on the –OH content of the starting materials. NH_3_ can be formed by deamination of –NH_2_. It is usually assumed that petroleum (oil and gas) can be formed by the thermal cracking of polymethyl chains of long-chain hydrocarbons and highly viscous organic matter (Fig. 1a). This explanation may be correct when the MC mixture (Fig. 1) is used as the starting material. However, the results of these additional experiments indicate additional sources of black oil, that is, simple mixtures of ethylene glycol, glycol acid, and glycerol (MC-1) and cyclohexyl phenyl ketone, 4’-cyclohexylacetophenone, acetamide, and urea (MC-2). Heating these molecules may cause polymerization at certain temperatures similar to the highly viscous organic matter; then, these polymers may decompose to form black oil at higher temperatures. Therefore, the possible sources of petroleum are more extensive than previously thought.

## Discussion

The atomic composition of the precometary-organic-matter analog used in the present study (MC) is H:C:N:O = 148:100:5:23 (O/C = 0.23, N/C = 0.01). The atomic compositions of organic particles from the comets 1 P/Halley and 81 P/Wild2 are H:C:N:O = 80:100:4:20 (O/C = 0.2, N/C = 0.04)^14^ and O/C = 0.2 − 0.6, N/C = 0.07 − 0.2^18^, respectively. The atomic compositions of organic particles from interplanetary dust particles and Antarctic micrometeorites, both of which may have originated from comets, are O/C = 0.49, N/C = 0.16^17^ and O/C = 0.27, N/C = 0.15^19^, respectively. If we compare these cometary organics with the starting material used in the present study, it is clear that cometary organics are rich in O and N, which shows their primitive nature. Even though we have no information concerning the content of -OH in actual cometary organics, we expect that the amount of water produced by heating organics will likely increase with increasing O content. Therefore, we suggest that the amount of water produced by the heating of actual precometary/cometary organics is likely larger than that obtained in the present experiments.

The present study suggests that liquid water can be formed via the heating of precometary organic matter to approximately 300 °C in the parent bodies of chondrites inside the snow line. Note that this temperature should be much lower on astrophysical time scales. The produced liquid water in the deeper parts of ordinary chondrite parent bodies can move upward and react with anhydrous silicates near the surfaces of their parent bodies to form hydrous silicates, as shown by Alexander *et al*.^53^. These parent bodies or their fragments may have served as a source of water for the terrestrial planets. In fact, some evidence for aqueous alteration has been found in primitive ordinary chondrites; for example, various hydrous silicates have been found in ordinary chondrites^53–56^. Even in highly heated ordinary chondrites, phosphate minerals have been found^57^, which might have formed via interactions with fluids during cooling. In addition, the D/H ratio of ordinary chondrites is extremely large^58^, suggesting isotope fractionation during dehydration. Very recently, water was directly detected in a *Hayabusa* sample collected from the S-type asteroid Itokawa^59^, which is equivalent to a parent body of ordinary chondrites. Further, oil produced in the deeper parts of parent bodies could move upward and mix with original precometary organics near the surfaces of such parent bodies. When this mixture is slightly heated in an open system, volatile components can vaporize while solid carbonaceous materials remain, as demonstrated experimentally^26^. In this way, insoluble organic matter, which has been found in some ordinary chondrites^55,60,61^, may form. Because the actual parent bodies are heterogeneous and more complex than those described above, water and subsequent hydrous silicates, and petroleum and subsequent insoluble organic matter, might form in localized areas. This inference is supported by the distribution of organic matter and hydrous silicates in carbonaceous chondrites^62–64^. The above observations of ordinary chondrites demonstrate that liquid water and subsequent hydrous silicates exist in the parent bodies of ordinary chondrites. Our experimental results highlight a possible new reservoir of water inside the snow line.

Geochemical evidence suggests that the initially accreted Earth was a highly reduced environment^9,10^. Most models^6–8^ of early stage water delivery to terrestrial-planet embryos cannot explain this highly reduced state because they assume that materials near Earth orbit were anhydrous silicates and OH (or H_2_O) introduced via various routes, and this combination does not produce a reduced state. In addition, if we assume that the materials near Earth orbit were completely dry, *i.e*., that only anhydrous silicates were present, it appears impossible to realize a highly reduced state. Therefore, the Earth’s materials have been simply assumed as mixtures of a highly reduced component and an oxidized component^65^, and the production mechanism of the highly reduced component has not been discussed. Experimental heating of the precometary-organic-matter analog in a vacuum demonstrated that water and other volatile gases evaporated at temperatures lower than 100 °C and that the remaining solid became carbonaceous matter^23,25^. From these results, we concluded that anhydrous silicate grains were likely covered by organic matter just inside the snow line or by carbonaceous materials far inside the snow line. Because the heating of carbonaceous materials can produce a highly reduced state^25^, the presence of such materials can overcome the “highly reduced state problem” for the early Earth. Material accreted later, but not as a late veneer, from ordinary and/or carbonaceous chondrites consist of anhydrous silicates, hydrous silicates, and small amounts of carbonaceous materials. These combinations can produce a more-oxidized state than that seen in the early stage. Earth’s water might have been delivered at this stage, even though the delivery mechanism is still under debate^4,11–13^. In this way, we can explain the change in the oxidation state from an initially reduced state to a final oxidized state. In late-stage water delivery models^11–13^, too much water is delivered to the terrestrial planets because it is delivered as hydrous silicates in carbonaceous chondrites from beyond the snow line. If water can form when precometary organics are warmed by secondary alteration in ordinary chondrites’ parent bodies, it could account for the abundant water brought to Earth by ordinary chondrites inside the snow line.

The formation of abiotic petroleum in some solar system bodies also needs to be considered. In the parent bodies of carbonaceous chondrites, the formation of soluble organic matter such as oil has been demonstrated experimentally via the high-temperature heating of insoluble organic matter with water^66,67^. The present study suggests the possibility that petroleum formation may have also occurred in the parent bodies of ordinary chondrites via the simple heating of precometary organics, as shown above. However, this petroleum may not remain at the present time because the oil may have changed to solid carbonaceous material (insoluble organic matter) following mild heating near the surfaces of the parent bodies, as discussed above. *Cassini* found several macromolecular organic compounds^68^ and hydrogen gas^69^ during its observations of Enceladus. Even though the formation mechanism of the organic compounds in Enceladus is still unclear, heating of precometary organics with ice may be included in the possibilities. Matson *et al*.^70^ estimated the maximum temperature of Enceladus as 1300 K. Therefore, we suggest that the reaction occurring in our experiments is similar to that occurring in the interior of Enceladus. Our density measurements of the oil and aqueous products suggest that an oil layer may exist between the icy crust and the liquid-water ocean of this satellite. Oil may also be concentrated beneath the basin known as Sputnik Planitia on Pluto because the bottom of the icy crust may have a concave shape^71^ similar to cap rocks on oil reservoirs on the Earth. Further, an oil layer beneath the icy crust could behave as a good thermal insulator to prevent Pluto from cooling, in addition to the recently proposed methane clathrate-hydrate thermally insulating layer^72^, because the thermal conductivity of crude oil^73^ is five times smaller than that of methane clathrate-hydrate. For the Earth, an abiotic petroleum formation mechanism has been discussed by Sephton and Hazen^74^, while Maurette *et al*. suggested the possibility that kerogen-rich micrometeories^75^ or cometary organics^76^ were a source of petroleum for the ancient Earth. The former possibility is supported by laboratory experiments^66,67^, and the latter may be supported by our present study.

## Methods

### Starting material

To create an analog of the organic material formed in molecular clouds and then processed in the solar system (Figs. 1 and SI 1), the chemical reagents were weighed and mixed in a mortar without any solvent in an N_2_ atmosphere.

### *In-situ* observations using a diamond anvil cell

To simulate the heating of precometary organic matter in a meteorite parent body that formed inside the snow line (<2.5 AU) up to several hundreds of degrees Kelvin^27–29^, we used an externally heated diamond anvil cell^47^ at the Institute for Planetary Materials, Okayama University. Diamond anvil cells are commonly used in high-pressure experiments. Because diamonds are transparent at visible, infrared, and X-ray wavelengths, both *in-situ* optical absorption spectroscopy and X-ray diffraction are possible. We placed the starting material into a 500-μm hole in a 125-μm-thick Ir gasket in air, together with a quartz crystal (Qz in Fig. 1a) for the pressure measurements. The pressures estimated using the method of Schmidt and Ziemann^77^ were 80 ± 50 MPa and 70 ± 50 MPa at 23 °C and 102 °C, respectively. However, at temperatures higher than 200 °C, we were not able to measure the pressure due to strong fluorescence from the organic matter. The thickness of the Ir gasket after the experiment was 122 μm, indicating that the pressure during the heating experiment was not very high. The temperature profile in the present experiment is shown in Fig. SI 2. During the experiment, we observed changes in the sample *in-situ* using an optical microscope and recorded these changes using a video recorder. We measured near-infrared spectra of 50 × 50-μm^2^ sample spots in transmission mode using a micro Fourier-transform spectrometer (Jasco IRT-7000/FTIR-6200). Spectra were acquired with 100 times accumulation at a resolution of 4 cm^−1^. We estimated the viscosity qualitatively from the movements and velocities of small spheres and interfaces.

### Heating experiment using an autoclave

We used an autoclave made of Hastelloy alloy (OM Labotec, MA type), which is shown in Fig. SI 3. After loading a 10-g sample into the autoclave in an N_2_ atmosphere, we evacuated the residual gas (primarily N_2_) using an oil-free scroll pump. We then heated the autoclave at a rate of 3 °C/min to 400 °C and maintained that temperature for 5 h. After heating, we cooled the sample to room temperature by turning off the electric power supply. The gas was recovered from the reactor to an evacuated gas bottle by opening a valve between the gas bottle and the reactor (see Fig. SI 3), and then closing a valve attached to the gas bottle. From the mass of the recovered products (oil and aqueous solution), we estimated the weight of the gas. We analyzed the recovered products (gas, oil, and aqueous products) using the following methods.

### Mid-infrared spectroscopy

We measured the mid-infrared absorption spectra of the starting material and the recovered oil and aqueous products using a Fourier transform infrared spectrometer (PerkinElmer, Spectrum One). Spectra were acquired with 100 times accumulation at a resolution of 4 cm^−1^ using an attenuated total reflection attachment.

### Aqueous product analysis via high-resolution mass spectrometry

We analyzed the aqueous product via high-resolution mass spectrometry using a Thermo Scientific Exactive with a mass resolution of *m/Δm* ~ 70,000 at a mass-to-charge ratio (*m*/*z*) of 200 (Thermo Fischer Scientific, Inc.). We dissolved a small aliquot of the sample in methanol and used flow injection to introduce approximately 5 μL of the solution into the mass spectrometer. We measured the mass spectrum in both the positive- and negative-electrospray ionization modes over an *m*/*z* range of 50–1000 at a spray voltage of ~3 kV. The capillary voltage and the temperature of the ion transfer were 25 V and 300 °C, respectively.

### Oil analysis

We analyzed the oil sample via MALDI-TOF mass spectrometry (MALDI-TOF-MS) using a TOF/TOF^TM^ 5800 system (SCIEX). We prepared the sample for the MALDI-TOF-MS analysis in accordance with the previously reported method^78,79^. Briefly, we dissolved an aliquot (0.1–0.3 mg) of the oil sample in 1 mL of tetrahydrofuran (THF) and mixed it with a matrix reagent (trans, trans-1,4-diphenyl-1,3-butadiene or 9-nitroanthracene). We then transferred the mixture to the MALDI target-plate cell using the water-spotting method. To remove all the THF from the sample, we allowed the entire sample to dry over a one-hour period. We conducted the MALDI-TOF-MS analysis over an *m/z* range of 0–2000. To confirm the repeatability of the data, we analyzed each sample five times at different spots.

We further subjected the recovered oil (0.5 mL) to column chromatography (5.0 cm × 10 mm i.d.) on a silica gel (Merck silica gel 60, 70–230 mesh, 5.0 g) with elution by *n*-hexane (30 mL) to isolate the saturated hydrocarbons and alkylated benzenes (fraction 1), with elution by dichloromethane/*n*-hexane (7:3 v:v, 14 mL) to isolate the aromatic compounds (fraction 2), and with elution by *n*-hexane/ethyl acetate (9:1 v:v, 10 mL) and ethyl acetate/methanol (1:1 v:v, 10 mL) to isolate the polar compounds (fraction 3). We performed GC-MS (HP6890-HP5973; Hewlett Packard) using a fused-silica DB-5 column (30 m × 0.25 mm i.d.; Hewlett Packard) with helium as a carrier gas at 0.6 mL/min. The oven temperature program was 40 °C (5 min) to 300 °C (maintained for 5 min) at 4 °C/min. We assigned compounds using the NIST Mass Spectral Search Program (National Institute of Standards and Technology), together with data from the literature^80,81^. We identified 1,4-Diisopropylbenzene by comparing the retention times with an authentic sample (Tokyo Chemical Industry Co.). The detailed analytical procedures are described in Asahina and Suzuki^82^.

### Gas analysis

We measured the composition of the recovered gases from the GC heating experiment using an instrument (7890 A: Agilent) equipped with a pulsed-discharge helium-ionization detector and a micropacked column containing ShinCarbon ST 80/100 (2.0 m × 1.0 mm i.d.; Shinwa Co.). We used ultra-high-purity helium as the carrier gas and introduced the gas at a constant rate into the GC column with a 10-μL sampling loop. The oven temperature program was 40 °C (3 min) to 300 °C (maintained for 15 min) at 15 °C/min. We identified compounds by comparing the retention times with those of reference standards in a gas mixture containing H_2_, CO, CO_2_, CH_4_, C_2_H_4_, C_2_H_6_, C_3_H_6_, C_3_H_8_, *i-*C_4_H_10_, and *n-*C_4_H_10_. The detailed analytical procedure is described in Saito *et al*.^83^.

## Supplementary information

Supplementary Information.

Video 1.

Video 2.

Video 3.

Video 4.

Video 5.

Video 6.

Video 7.

## Data Availability

The data that support the findings of this study are available from the corresponding author upon reasonable request.
