# Supplementary material for: Precometary organic matter: A hidden reservoir of water inside the snow line
Source: Sci Rep. 2020 May 8;10:7755. doi: 10.1038/s41598-020-64815-6 (PMC7211008; doi:10.1038/s41598-020-64815-6)
Supplement: Supplementary file 1 — Supplementary Information. [file 41598_2020_64815_MOESM1_ESM.docx]

**Supplementary Information**

**Precometary organic matter: A hidden reservoir of water inside the snow line**

Hideyuki Nakano^1^†, Naoki Hirakawa^1^‡, Yasuhiro Matsubara^1^, Shigeru Yamashita^2^, Takuo Okuchi^2^, Kenta Asahina^3^, Ryo Tanaka^4^#, Noriyuki Suzuki^4^, Hiroshi Naraoka^5^, Yoshinori Takano^6^, Shogo Tachibana^7,8^, Tetsuya Hama^9^, Yasuhiro Oba^9^, Yuki Kimura^9^, Naoki Watanabe^9^, & Akira Kouchi^9^*

**Figure SI 1.** Composition of simple mixtures **(a)** including –OH and –COOH and **(b)** of amides and ketones without –OH. The ratios of each compound are the same as those in Fig. 1.

**Figure SI 2.** Temperature profile of the heating experiment using a diamond anvil cell. The pale-blue squares and red circles indicate the points at which we took photomicrographs (Fig. 2) and measurements of the near-infrared spectra (Fig. 3a), respectively. The blue lines show the video-recording durations of Videos SI 1–7.

**Figure SI 3.** The autoclave used in the present study.

**Figure SI 4.** MALDI-TOF spectrum of the recovered oil product from the heating of MC at 400 °C over an *m/*z range of 200–800.

**a b**

**
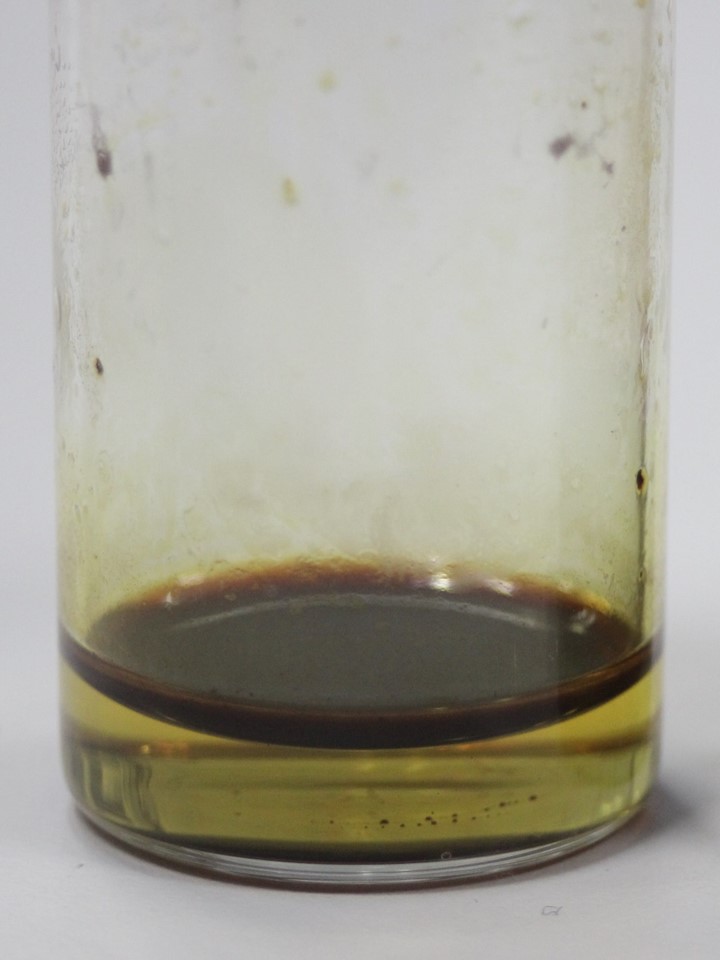

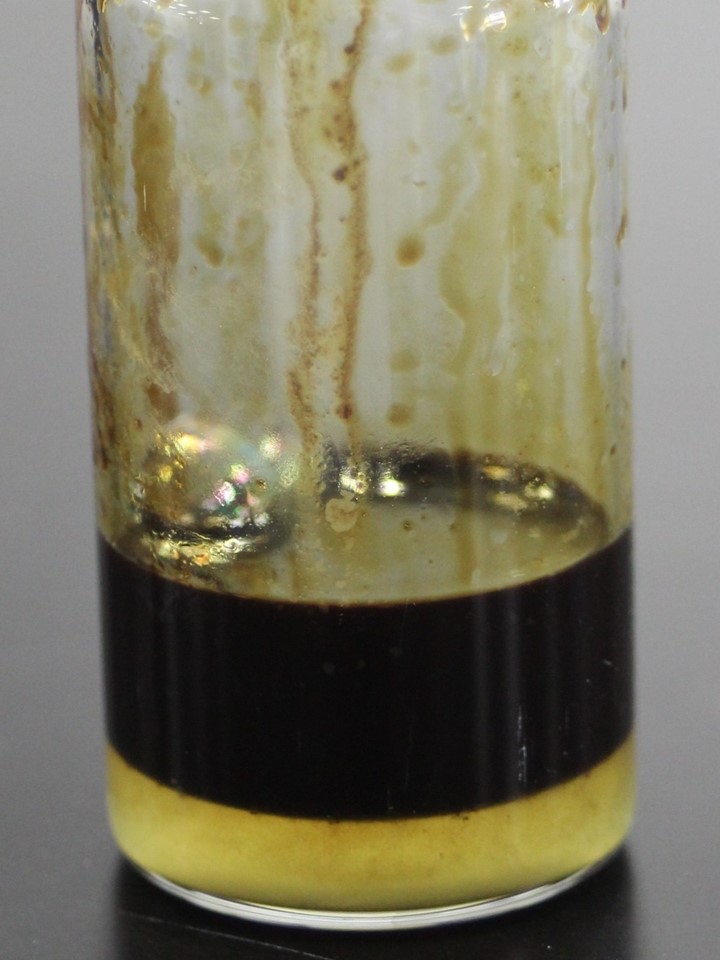
**

**Figure SI 5.** The black oil and transparent liquid recovered from the heating experiments of **(a)** MC-1 and **(b)** MC-2, respectively, at 400 °C. The diameter of the sample bottles is 30 mm.

**Figure SI 6.** Mid-infrared absorption spectra of pure water (**a**, reference, gray), the recovered liquid product obtained from MC-1 (**b**, blue), and the recovered liquid product obtained from MC-2 (**c**, red).

Video SI 1.

Video recorded from 24 °C to 100 °C, corresponding to V-1 in Fig. SI 1 and reproduced by quad-speed.

Video SI 2.

Video recorded from 100 °C to 200 °C, corresponding to V-2 in Fig. SI 1 and reproduced by quad-speed.

Video SI 3.

Video recorded from 200 °C to 250 °C, corresponding to V-3 in Fig. SI 1 and reproduced by quad-speed.

Video SI 4.

Video recorded from 250 °C to 300 °C, corresponding to V-4 in Fig. SI 1 and reproduced by quad-speed.

Video SI 5.

Video recorded from 300 °C to 350 °C, corresponding to V-5 in Fig. SI 1 and reproduced by quad-speed.

Video SI 6.

Video recorded from 350 °C to 400 °C, corresponding to V-6 in Fig. SI 1 and reproduced by quad-speed.

Video SI 7.

Video recorded from 400 °C to 300 °C, corresponding to V-7 in Fig. SI 1 and reproduced by quad-speed.
